# Circular RNA Expression: Its Potential Regulation and Function in Abdominal Aortic Aneurysms

**DOI:** 10.1155/2021/9934951

**Published:** 2021-06-29

**Authors:** Yanshuo Han, Hao Zhang, Ce Bian, Chen Chen, Simei Tu, Jiahui Guo, Yihao Wu, Dittmar Böckler, Jian Zhang

**Affiliations:** ^1^School of Life and Pharmaceutical Science, Dalian University of Technology, Dalian, China; ^2^Department of Cardiovascular Surgery, The General Hospital of the PLA Rocket Force, Beijing Normal University, Beijing, China; ^3^School of Biomedical Sciences, The University of Queensland, Brisbane, Australia; ^4^Department of Vascular and Endovascular Surgery, University of Heidelberg, Heidelberg, Germany; ^5^Department of Vascular Surgery, The First Hospital, China Medical University, Shenyang, China

## Abstract

Abdominal aortic aneurysms (AAAs) have posed a great threat to human life, and the necessity of its monitoring and treatment is decided by symptomatology and/or the aneurysm size. Accumulating evidence suggests that circular RNAs (circRNAs) contribute a part to the pathogenesis of AAAs. circRNAs are novel single-stranded RNAs with a closed loop structure and high stability, having become the candidate biomarkers for numerous kinds of human disorders. Besides, circRNAs act as molecular “sponge” in organisms, capable of regulating the transcription level. Here, we characterize that the molecular mechanisms underlying the role of circRNAs in AAA development were further elucidated. In the present work, studies on the biosynthesis, bibliometrics, and mechanisms of action of circRNAs were aims comprehensively reviewed, the role of circRNAs in the AAA pathogenic mechanism was illustrated, and their potential in diagnosing AAAs was examined. Moreover, the current evidence about the effects of circRNAs on AAA development through modulating endothelial cells (ECs), macrophages, and vascular smooth muscle cells (VSMCs) was summarized. Through thorough investigation, the molecular mechanisms underlying the role of circRNAs in AAA development were further elucidated. The results demonstrated that circRNAs had the application potential in the diagnosis and prevention of AAAs in clinical practice. The study of circRNA regulatory pathways would be of great assistance to the etiologic research of AAAs.

## 1. Introduction

Abdominal aortic aneurysms (AAAs) frequently induce cardiovascular death among the elderly male population within various European and Asian countries [1–3]. AAAs are featured by permanent expansion and weakening of a localized abdominal aorta [4–6]. There are symptomatic, asymptomatic, and ruptured AAAs clinically [7]. As estimated by a UK study, 1.5% of its population has an AAA greater than 30 mm in size [8]. Besides, a USA multicenter aneurysm screening study has suggested that 4.6% of its population aged 65-74 suffers from an AAA [9]. In Sweden, the incidence of the AAA among male population aged above 65 is reported to be 1.8% [10]. Because of the lack of effective surgical methods and unpredictability of the disease, continuous enlargement of the aortic wall will lead to the wall rupture and serious bleeding, resulting in a death rate as high as 80% [11]. A majority of AAA cases are progressive and asymptomatic, which are usually discovered accidentally by the diagnostic imaging of other diseases. The survival of AAA patients after diagnosis has improved, but its morbidity and mortality rates show an ascending trend [3]. Generally, the AAA-related mortality cannot be affected by AAA screening, but may be lowered by other factors like smoking less [12].

Conventionally, AAAs are mostly treated with elective retroperitoneal or open transperitoneal surgery [13], but now, the endovascular repair (EVAR) is recommended as a better alternative to open surgerical repair [14–16]. Currently, no curative treatment is available for restricting AAA development and preventing AAA rupture [17], and the only strategy is to continuously monitor the aneurysm size before surgery [18].

The pathogenic mechanism of AAAs is complicated and involves multiple factors. Previous studies suggest that AAAs are associated with the weakened defective adventitial/medial arterial layers, like fibroblasts and smooth muscle cells (SMCs) [19–22]. Recently, research on human tissue and animal models indicates that the AAA occurrence arises from the dynamic vascular remodelling [23, 24]. Additionally, the critical pathological features of AAAs are oxidative stress (OS), vascular inflammation, aortic wall thinning due to the loss of vascular smooth muscle cells (VSMCs), and aortic extracellular matrix (ECM) decomposition [22, 24].

Due to strong gene expression regulation effects, epigenetic alterations, such as histone modification, DNA methylation, and noncoding RNA (ncRNA) modification [25–27], have been increasingly acknowledged as an importance contributor to AAA development. It has been proven that epigenetic modifications take place in the early embryogenesis and primordial cell development processes, but it is of great significance to explore the effects of these alterations in “later life.” In such, “later life” epigenetic modifications caused by the dietary intervention were analyzed [28]. Generally, ncRNAs can be divided into small ncRNAs and long ncRNAs (lncRNAs) according to the arbitrary threshold size of 200 nucleotides (nt). Among small ncRNAs, microRNAs (miRNAs) with a length of about 22 nt have been extensively investigated. By contrast, there is less studies on the functions of lncRNAs with a length of over 200 nt. Although ncRNAs have been demonstrated to regulate the interactions and activities of fibroblasts, vascular inflammatory cells, endothelial cells (ECs), and SMCs, the key factors resulting in AAA occurrence remain unidentified. According to a new eukaryotic gene expression feature discovered in 2012, circRNAs are ubiquitously expressed in genes previously considered to express linear ncRNAs or messenger RNAs (mRNAs) only. circRNAs are RNA molecules with a circular and covalently closed structure, which usually consist of exon sequences and can be spliced at the typical splice sites.

In particular, many articles have demonstrated the important roles of ncRNAs in cardiovascular diseases (CVDs), such as aortic dissection and AAAs. Meanwhile, circRNAs are also considered to exhibit certain effects on some CVDs, but their expression levels and roles in AAAs still remain unclarified.

In the present work, the roles of circRNAs in the occurrence and development of AAAs were illustrated, and their significance in treating AAAs was discussed. In addition, the regulation effect of circRNAs and their corresponding target genes on AAAs and the underlying mechanisms were also investigated. The ncRNAs that were previously recognized to be involved in several processes leading in AAAs were highlighted. The in-depth investigation on such circRNAs will shed lights on the prevention and treatment of AAAs, and these circRNAs may be used as candidate prognostic biomarkers and therapeutic targets for the prediction of the AAA incidence and the evaluation of patient prognosis.

## 2. Genetic Causes of AAA Pathogenesis

### 2.1. Mendelian Causes

A Mendelian factor that causes AAAs often refers to single-gene mutations [29]. Marfan syndrome can also induce AAAs, which is often accompanied by a family history. Marfan syndrome arises mainly from FBN1 gene mutation [30], accounting for an autosomal dominant genetic disorder. The TGF*β*R1, TGF*β*R2, and TGF*β*R3 gene mutations promote the production of matrix metalloproteinases (MMPs) and eventually lead to medial degradation [31]. Other syndromes can provoke the occurrence of aortic root aneurysms. In addition, some diseases derived from single-gene mutations affect ECM components of the aortic wall. The Ehlers Danlos syndrome, for instance, originates from COL3A1 gene mutation [32]. AAAs may be detected in cases who develop autosomal recessive disorders, such as pseudoxanthoma elasticum and homocystinuria, which stem from ABCC6 and CBS gene mutations, respectively [29].

### 2.2. Non-Mendelian Causes

There are various non-Mendelian factors leading to AAAs, including the ECM, endothelial cell (EC) specification, SMCs differentiation, inflammation, and cell adhesion.

#### 2.2.1. ECM

The ECM is a complex network of macromolecular substances that supports and connects tissue as well as mediates tissue generation and cell physiological activities. The proteins of the Matrix Metalloproteinases (MMP) family can regulate the ECM turnover, which is related to the formation of genes that encode MMP and ECM components in AAAs. Besides, upregulated MMP-2 and MMP-9 levels can be detected within human AAA tissue [33, 34]. Moreover, the single-nucleotide polymorphisms (SNPs) rs3025058 in MMP-3 and rs2252070 in MMP-13 are suggested to lead to a higher AAA risk [35]. Inhibiting the ablation of MMP-3 can promote aneurysm formation. Additionally, the G allele of rs2252070 also contributes to a higher AAA risk [36].

#### 2.2.2. SMC Differentiation

AAAs are related to rs1795061 localized at around 40 kb upstream of the SMYD2 transcription start site [37]. SMYD proteins belong to the lysine methyltransferases, and they play a vital part in the regulation of cardiac and skeletal morphogenesis [38]. According to a recent report, SMYD2 is possibly associated with the pathogenic mechanism of AAAs [39]. Importantly, Toghill et al. evaluated DNA methylation within VSMCs collected from AAA patients using targeted bisulphite next-generation sequencing (NGS) for the first time. They found evident DNA hypomethylation within the promoter region of SYMD2 gene among AAA cases and a close association between the average methylation degree of CpGs and gene expression levels [40]. Typically, methylation of the SMYD2 promoter region may be related to AAA development, rather than its progression [40].

#### 2.2.3. EC Specification

In the study of Jones et al., the SNP rs2836411, an AAA risk variation localized at 21q22.2 in the intrinsic region of ERG gene, was detected [37]. ERG can encode one transcription factor (TF) existing in ECs and hematopoietic cells (HPCs) under normal conditions. ERG plays a part in the vascular development mediated by the vascular endothelial growth factor (VEGF)/mitogen-activated protein kinase (MAPK) and also regulates new vessel formation [41]. The minor (T) allele of rs2836411 correlates with a greater risk of AAAs in several populations [42].

#### 2.2.4. Inflammation

CD4^+^T cells are found to accumulate on the aneurysm wall, and AAA-related SNPs are detected in some human genes encoding such critical inflammatory components. AAA patients show an increased circulating IL-6 level. IL-6 is secreted by the aneurysm, and its expression level is related to the aneurysm surface area. Angiotensin II can affect AAAs by acting on the IL-6 signaling pathway in mice.

## 3. Epigenetic Regulation of AAAs

Epigenetic modifications are induced by developmental or environmental factors. They do not change the genetic code but can regulate the tissue- or context-specific expression of information encoded in DNA [43, 44]. Traditional views hold that epigenetic markers are stable, which may be transmitted to progeny, and control the steady differentiation of different types of cells with significantly diverse gene expression profiles [45]. On the whole, epigenetic alterations are divided into 3 major types: (1) DNA methylation, (2) histone modifications, and (3) ncRNAs. Of them, ncRNAs will not bring about heritable alterations, but they are usually deemed as epigenetic mechanisms due to their vital regulating functions in the genome nonprotein-coding regions. A small number of studies have revealed the important role of the above-mentioned three categories of epigenetic modifications in AAA occurrence [25–27, 40, 46, 47].

### 3.1. DNA Methylation in AAAs

DNA methylation, one of the potent epigenetic mechanisms, is crucial in preserving the DNA structure, deactivating the X chromosome and maintaining chromosome stability. Besides, DNA methylation also modulates components (retrotransposons and transposons) and regulates gene levels [48]. It is claimed that some key processes contributing to AAA occurrence may be affected by DNA methylation. Generally speaking, DNA methylation is catalyzed by the DNA methyltransferases (DNMTs), including DNMT1 (maintenance enzymes) and DNMT3 (de novo enzymes). Researchers are focusing on DNA methylation in T lymphocyte, and the analysis of different types of vascular cells, such as ECs, SMCs, and adventitial cells, may provide more targets specific to aneurysms [47]. Increasing evidence suggests that T cell dysfunction, especially the reduced suppression of CD4^+^ CD25^+^ regulatory T cells, induces AAA occurrence [46]. Apart from DNA hypomethylation, DNA hypermethylation in certain gene promoter regions also contributes to AAA occurrence. This opinion is addressed in a recent article, which evaluates the global methylation level in AAA patient-derived peripheral blood mononuclear cells (PBMCs) and compares it with that in PBMCs from normal subjects [40]. The study conducted by Skorvanova et al. revealed no association of AAA development with DNA methylation of gelatinases and their tissue inhibitors (e.g., MMP2, TIMP2, TIMP1, and MMP9) [49].

### 3.2. Histone Acetylation in AAAs

Gene activation and inactivation depend on specific signatures of histone modifications in critical enhancer or promoter gene regions. Such modifications are harbored in histone deacetylases, histone acetylases, together with methyltransferases [50]. The findings of our work show that the abnormal epigenetic modifications in AAAs are the changed expression of lysine [K] histone acetyltransferases (KATs) and related histone acetylation. The above results shed novel lights on the pathogenic mechanism of AAAs [26]. As suggested in another study, the levels of some histone deacetylases (HDACs) in AAA mice and ApoE^−/−^AAA mice infused with Angiotensin (Ang) II are higher than those in normal controls [51]. As a class III HDAC, Sirtuin 1 (SIRT1) is also studied for its role in AAA occurrence and the mechanisms underlying vascular inflammation and aging. The result suggests that SIRT1 in VSMCs provides a new therapeutic target for preventing AAA occurrence [52].

### 3.3. ncRNAs in AAAs

It has been established that over 97% of the genome can encode the noncoding transcripts, most of which can be processed into short ncRNAs (such as miRNAs) and lncRNAs. There are various factors leading to AAAs, and the pathology and pathogenic mechanisms of AAAs at the molecular level remain largely unclear. Therefore, it will become a novel research direction to investigate the AAA etiology at the epigenetic level. Studies have shown that ncRNAs take part in the AAA genesis. Many ncRNAs are differentially expressed in AAA patients and regulate gene expression at transcriptional and posttranscriptional levels. The regulatory pathways of some ncRNAs have also been confirmed, which is conducive to the study of other molecular mechanisms [27, 53–56].

#### 3.3.1. miRNAs in AAAs

MiRNAs are short (21-23-nt-long) RNA molecules existing in eukaryotes, capable of regulating the expression of other genes [57]. They are ncRNAs and control posttranscriptional expression by specifically binding with mRNAs [58]. In general, negative regulation is mainly achieved by the degradation of mRNAs or inhibition of mRNAs expression. MiRNAs can be regulated by a variety of approaches. A miRNA may possess several target genes, while several miRNAs may modulate the same target gene. MiRNAs adjust target gene expression of one or more genes through forming complicated regulatory networks [59, 60]. MiRNAs are closely associated with the AAA pathogenic mechanism [61]. In AAA patients, miRNAs affect the pathogenesis of AAAs by forming networks to regulate the ECM turnover, MMP family, different inflammatory components, and vascular smooth muscle development [62–65].

The most typical type of miRNAs is miRNA-29 family members, which promote the AAA formation by regulating fibrosis and the ECM. miRNA-29 inhibits the expression of several ECM proteins and antiapoptotic factors in SMCs, thereby boosting the aneurysm generation [66, 67]. Studies have confirmed that AAAs with overexpressed miRNA-29b are more prone to rupture. Reducing the miRNA-29b levels can lower the probability of AAA rupture. Upregulation of miRNA-21 can be detected in AAA patients and mouse models. Overexpressed miRNA-21 can downregulate the phosphatase and tensin homolog (PTEN) levels, thereby inhibiting the viability of SMCs and maintaining vessel wall stability in mouse models with elastin degradation-induced aneurysms [68]. Moreover, Wang et al. have observed that the anti-miRNA-21 drug-eluting stent successfully prevents in-stent myointimal hyperplasia in a humanized rat model, further highlighting the complex roles of miRNA-21 in various vascular pathologies [69]. Furthermore, a decline in miRNA-33 leads to attenuated p38 and JNK (c-Jun N-terminal kinase) signals as a result of increased ABCA1 expression, the inhibition effect of CaCl_2_ and Ang II-induced aneurysm formation in mice [70]. In conclusion, the expression level of miRNAs is tightly bound to the AAA development. Therefore, it is of great significance to study the pathogenesis of AAAs.

#### 3.3.2. lncRNAs in AAAs

lncRNAs are a type of ncRNAs longer than 200 nt without the protein encoding function, and they are involved in regulating numerous processes [71]. In recent years, many research groups have been devoted to the study of lncRNAs and their pathological function [72]. However, a lot of efforts and in-depth exploration are still required in this field. lncRNAs serve as a gene domain and TF scaffold, guiding the transcription complex of activators and inhibitors to the regulatory region to regulate transcription [73, 74]. One lncRNA associated with the AAA is H19. The H19 level in AAA samples is higher than that in normal controls, and inhibiting the expression of H19 arrests the growth of AAAs [53]. H19 seems to affect the pathogenesis of AAAs through inflammation [55]. Another study has shown that PVT1, an lncRNA, is upregulated in AAA patients. Overexpression of PVT1 promotes apoptosis of VSMCs and degradation of the ECM [75], and PVT1 knockout in vitro notably reduces the incidence of AAAs. It further confirms that PVT1 promotes the formation of AAAs. lncRNAs can also affect AAAs via acting on the development and differentiation of SMCs. The representative example is lnc-Ang362, which enhances the proliferation of VSMCs [76]. Moreover, relevant studies have proven that SMILR can regulate the migration and proliferation of VSMCs, and SMILR knockout restrains the proliferation of SMCs [77], indicating that lncRNAs also play an important part in AAA occurrence and progression. Decreased plasma levels of SMILR have been observed in TAA cases [78].

#### 3.3.3. circRNAs in AAAs

**Figure 1:**
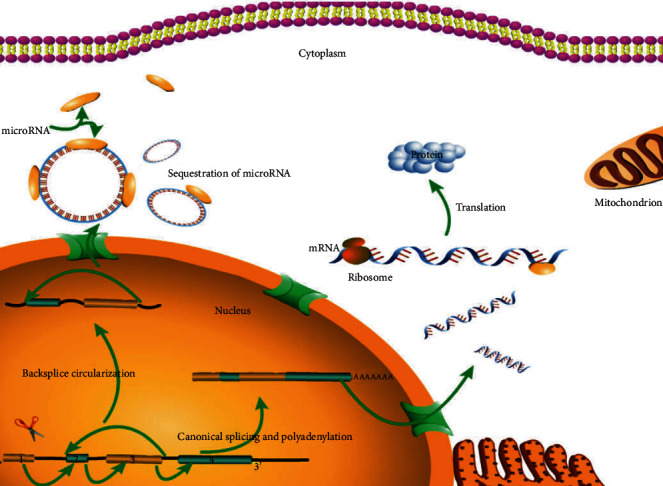
Formation and mechanism of RNA in cells. The formation process and mode of action of circRNAs and mRNAs in cells.

Like lncRNAs, circRNA transcripts have been a recent addition to the functionally relevant ncRNAs in our genomic landscape. They have long been disregarded since their discovery in the 1990s due in part to a limitation in their detection method, but the emerging novel bioinformatics and deep RNA sequencing approaches provide the foundation for circRNA research [79]. Recent studies suggest that there are many endogenous circRNAs in mammalian cells, and some of them show high abundances and evolutionary conservation. circRNAs initially have been shown to mediate miRNA functions (e.g., via sponging) and control important events in transcription (e.g., RNA folding and endonuclease protection) [80]. At present, circRNAs are proven to be stable, endogenous and functional ncRNAs abundant in mammalian cells [81]. They may be generated through directly ligating the 3′ and 5′ ends of linear RNAs by means of back-splicing [82]. The downstream 5′ splice site (donor) will bind to the upstream 3′ site (acceptor) or serve as the intermediate during RNA processing (Figure 1). Not every circRNA possesses many miRNA binding sites, so it remains controversial whether miRNAs are suppressed by circRNAs.

#### 3.3.4. Bibliometrics of ncRNAs in AAAs

**Figure 2:**
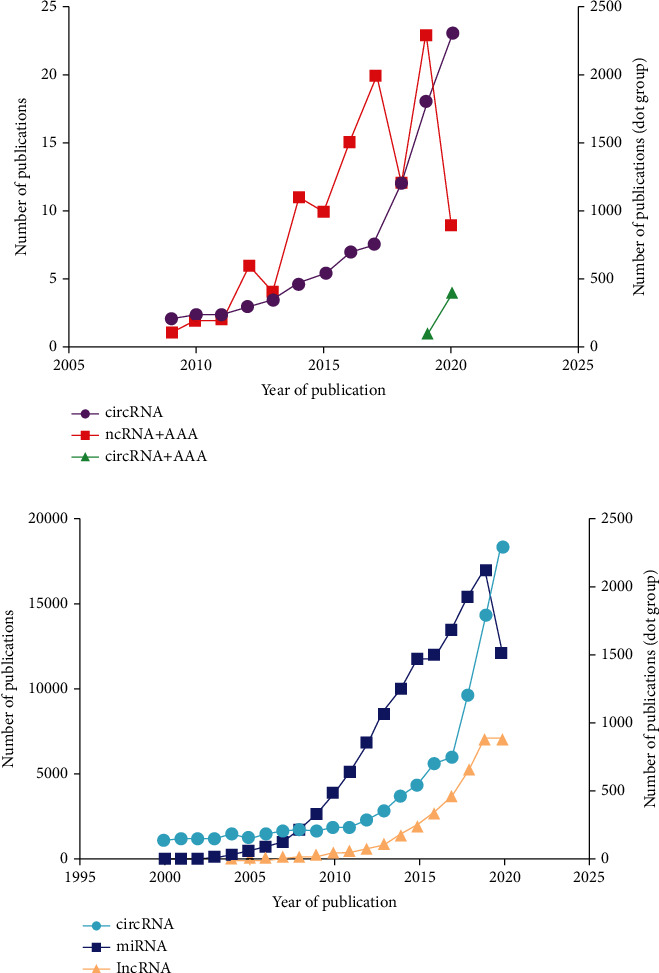
Statistics of AAA and ncRNA papers published. (a) The relationship between “circRNA, ncRNA, and abdominal aortic aneurysm” and “published volume and year of publication” (b) The relationship between the number of “circRNA, miRNA, mRNA” and “the year of publication”.

Firstly, we explored the relationship between publication volume and time in Figure 2(a), the abscissa is the time series, and the ordinate is publication volume. The purple dot represents the annual publication volume for publication of circRNAs, corresponding to the right ordinate axis. Meanwhile, the red block represents the annual publication volume of “ncRNAs and AAAs,” corresponding to the left ordinate axis. Moreover, green triangle represents the annual publication volume of “circRNAs and AAAs” and also corresponds to left ordinate axis. From 2009 to 2020, the number of papers published on “ncRNAs and AAAs” has increased year by year. There are large quantities of papers on circRNAs, up to more than 2000 papers, but few about the relationship between “circRNAs and AAAs,” but they are generally close. The past few years have witnessed an ascending trend (Figure 2(a)).

Then, we also explored the relationship between publication volume and time for ncRNA in Figure 2(b). The cyan dot represents the annual publication volume of circRNAs corresponding to the right ordinate axis, the blue block represents the annual publication volume of miRNAs corresponding to the left ordinate axis, and the orange triangle represents the annual publication volume of lncRNAs corresponding to left ordinate axis. From 2000 to 2020, there are great many papers published on ncRNAs (circRNAs, lncRNAs, and miRNAs) annually, and the total number is still climbing. The miRNA publications account for the largest part of the total number of published papers, up to more than 15000 publications, while circRNA publications take up the smallest share (Figure 2(b)). The above data imply that the relationship between “circRNAs and AAAs” may become a research hotspot and the international research direction in the future.

**Figure 3:**
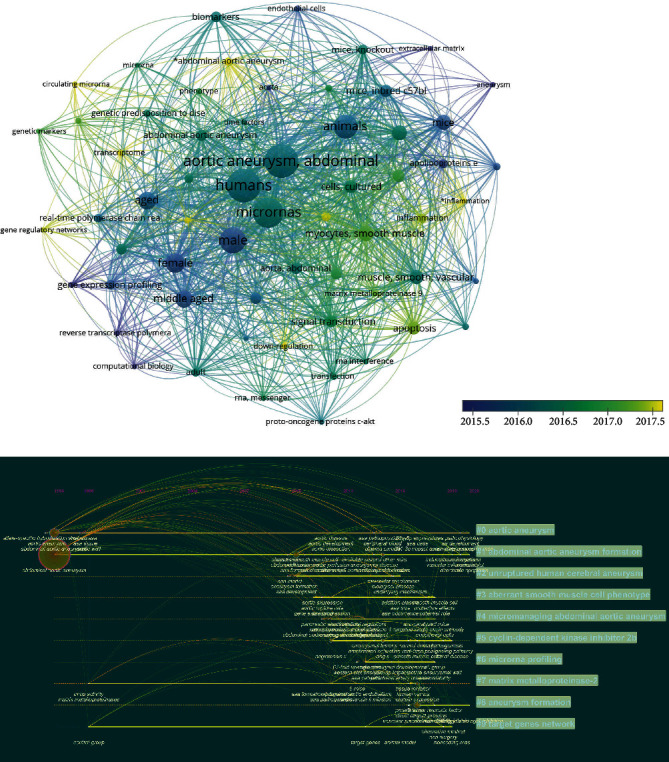
Bibliometric analysis of keywords. Keyword cluster analysis, visual analysis based on the year when the keyword appears, so as to make (a) network drawing and (b) timeline graph production.

Keyword analysis is relatively essential to the research of the entire paper. It can help us to figure out the research direction.  Figure 3(a) is the tag view of keywords, which reflects the timing of keywords and the relationship network diagram of cluster analysis. The size of the dot indicates the frequency of occurrence, and the colour of the dot represents the cluster and the time of occurrence. The curve describes the interrelationship. The disease formation, target, and regulation mode can be roughly acquired through cluster analysis. It can be seen from the time sequence that lncRNAs, AAAs, and circRNAs are newly emerging keywords in recent years.  Figure 3(b) is a timeline view of analyzing keywords from 1996 to 2020.

**Figure 4:**
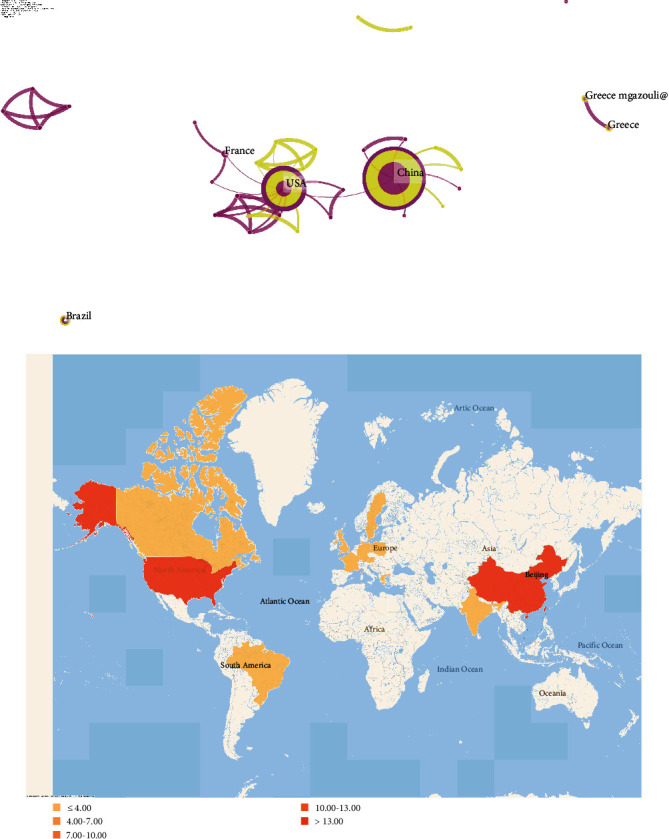
The analysis of total country output based on ncRNA research. (a) Network map of countries/regions engaged in ncRNA research (b) World map of total country output engaged in ncRNA research.

The classification method is to name the index terms in the search keywords. We can get ten clusters with an order from #0 to #10. The smaller the number # is, the more keywords the cluster contains. Each cluster is composed of multiple closely related words. The largest cluster is “aortic aneurysm” (#0), followed by “abdominal aortic aneurysm formation” (#1). A module clustering value (*Q* value) greater than >3 generally means a significant clustering structure. In the figure, modularity *Q* = 0.684.  Figure 4(a) is a network diagram obtained based on the number of papers published and cooperative relationships in each country, showing the interconnection between countries. The heat map in Figure 4(b) presents the number of national papers published on the world map.

## 4. Verified circRNAs in AAAs

Data on circRNAs in aneurysms are scarce, but abnormal expression of circRNAs in aneurysms has been observed in emerging studies. However, the functional role(s) of these circRNAs in AAA development in animal models and their therapeutic potential are yet to be elucidated.

### 4.1. circRNA Microarray Profiling of Human AAAs

Zhou et al. from China observed differential expression of circRNAs in AAA samples and controls, so they subsequently conducted high-throughput sequencing to determine the circRNA expression patterns in four pairs of aortic samples, including four consecutive AAA cases undergoing open surgery and four brain-dead heart-beating organ samples [83]. Finally, a total of 411 differentially expressed circRNAs were detected in AAA samples, including 145 upregulated circRNAs and 266 downregulated circRNAs. Six abnormally expressed circRNAs, namely, hsa_circ_0070382 (AFF1), hsa_circ_0060063 (UQCC1), hsa_circ_0028198 (ANAPC7), hsa_circ_0027446 (HMGA2), hsa_circ_0002168 (TMEM189), and hsa_circ_0005360 (LDLR), were then screened out for RT-PCR analysis. Among the six circRNAs, 2 were upregulated, and 4 were downregulated. According to a population-based genome-wide association study, LDLR is the parental gene of hsa_circ_0005360, and its variant is related to AAAs [84]. Additionally, LDLR-deficient mice infused with Ang II infusion are extensively used as AAA animal models [85–87]. Given the alternative transcription of hsa_circ_0005360 in the LDLR exons, hsa_circ_0005360 possibly plays a vital part in the pathogenic mechanism of AAAs. In the meantime, the interaction networks of circRNAs/miRNAs were also predicted by computational analysis (Table 1).

**Figure 5:**
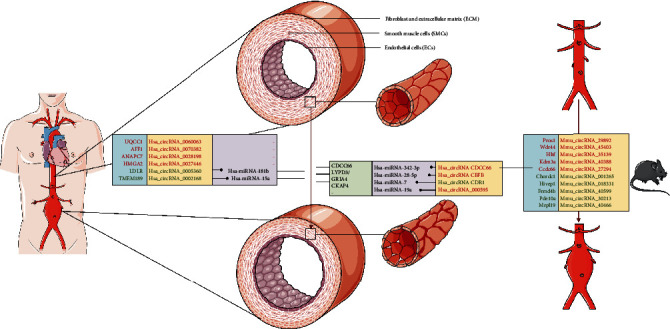
Several circRNA mediated signaling pathways in AAA. In human and mouse abdominal aortic aneurysms, the plan view of the abdominal aorta and several circRNA mediated signal pathways.

As indicated in the interaction networks of circRNAs/miRNAs, hsa_circ_0002168 and hsa_circ_0005360 contain one binding site for miR-15a and miR-181b, respectively. It is evident that overexpressed miR-181b in AAA patients downregulates elastin and the MMP-3 tissue inhibitor, thereby promoting AAA development [88]. As for miR-15a, it negatively regulates CDKN2B expression and thereby promotes VSMC apoptosis, possibly resulting in AAA pathogenesis [89]. Nonetheless, the hsa_circ_0002168/miR-15a and hsa_circ_0005360/miR-181b axes should be further validated in AAAs by more studies (Figure 5).

### 4.2. circRNA Microarray Profiling of Mouse AAAs Induced by Ang II

Wang et al. collected two samples from each of the AAA and control groups and analyzed the circRNA expression profiles. The AAA samples of C57BL/6J male mice were treated with Ang II and 3,4-benzopyrene (BAP), and the circRNA expression in these AAA samples was compared with that in the control group [90]. The qRT-PCR results showed that 271 circRNAs showed upregulation while 142 circRNAs were downregulated (Table 1). After predicting the related regulatory pathway, the authors mapped the downregulated mRNAs into 7 pathways, including apoptosis. It should be noted that apoptosis is well-received as a critical biological process of VSMCs in AAAs, and VSMC apoptosis can also be detected in AAAs induced by Bap/Ang II. In line with the above results, Wang et al. examined the competitive endogenous RNA (ceRNA) mechanism related to certain apoptotic circRNAs. For instance, two miRNAs (mmu-let-7a-2-3p and mmu-miR-199a-3p) of differentially expressed mmu_circRNA_001265 and their response elements were predicted. Another study suggests that lncRNA H19 causes aneurysms partially through endogenously competing with let-7a miRNA for inducing transcription of the target gene (IL-6) [55].

### 4.3. Examples of Several circRNA-Guided Signaling Pathways in AAAs

#### 4.3.1. circRNA CCDC66

To prove the role of circRNA CCDC66 in the pathogenesis of AAAs and identify its corresponding pathway, Yang et al. carried out a study and found that circCCDC66 played a role in the proliferation of VSMCs [91]. Through further investigation, they discovered that circCCDC66 promoted the expression of CCDC66, and CCDC66 suppression had the same effect as circCCDC66 deletion on the development and apoptosis of VSMCs. Moreover, the RNA pull-down test results suggested that the content of circCCDC66 and CCDC66 was the highest in the miR-342-3p group, indicating that circCCDC66 influenced CCDC66 through miR-342-3p. Taken together, it is concluded that overexpression of CCDC66 causes AAAs through the circCCDC66/miR-342-3p/CCDC66 pathway (Figure 5).

#### 4.3.2. circRNA CBFB

Findings of this study reveal that miR-28-5p, circCBFB, GRIA4, and LYPD3 are involved in AAAs. circCBFB serves as the miR-28-5p sponge, capable of regulating the proliferation and apoptosis of VSMCs in a LYPD3/GRIA4-dependent manner [92]. With regard to the relationship between circCBFB and AAAs, it is established previously that the expression of miR-28-5p increases in AAAs. As reported by Yue et al., miR-28-5p promotes VSMC apoptosis and suppresses their proliferation, thereby contributing to AAA occurrence [92]. Moreover, bioinformatic analysis verifies LYPD3 and GRIA4 as the potential miR-28-5p target genes. The mechanical experiments of this study suggest that miR-28-5p targets GRIA4 and LYPD3 and restrains their expression in VSMCs. Additionally, functional analysis proves that GRIA4 and LYPD3 deficiency promotes the apoptosis of VSMCs. More innovative strategies are required to identify the role of the circCBFB molecule in AAAs. Based on the above analysis, it can be concluded that circCBFB induces cell apoptosis and AAA formation through the circCBFB/miR-28-5p/GRIA4/LYPD3 pathway (Figure 5).

#### 4.3.3. circRNA CDR1

There are over 70 miR-7 binding sites in the cerebellar degeneration-related protein 1 antisense RNA (CDR1as), and these sites regulate the effect of CDR1as on the target gene expression [93]. According to recent research, the expression levels of CDR1as and CKAP4 (the estimated miR-7 target) are lower in AAA cases than those in normal controls [94]. Zhao et al. suggested that the CDR1as/miR-7/CKAP4 axis plays a role in VSMCs of AAA cases and that CDR1as upregulation may curb the expression of miR-7. What is more, CDR1as upregulation enhances the CKAP4 level, promotes VSMC proliferation, and suppresses VSMC apoptosis, thereby resulting in VSMC remodeling along with AAA progression. Such novel mechanism offers insights into AAA treatment approaches (Figure 5).

#### 4.3.4. Hsa_circ_000595

Through screening circRNAs in tissue specimens from AAA patients, Zheng et al. observed increased expression of hsa-circ-000595 in diseased specimens [95]. A similar pattern was found in hypoxic aortic SMCs, and the knockdown of hsa-circ-000595 reduced SMC apoptosis. Besides, they also found that miR-19a might serve as a potential downstream target of hsa-circ-000595. Hsa-circ-000595 on the chromosome 14 can modulate the miR-19a activity and acts on AAAs through suppressing cell apoptosis [95].

## 5. Potential circRNAs in AAAs

**Figure 6:**
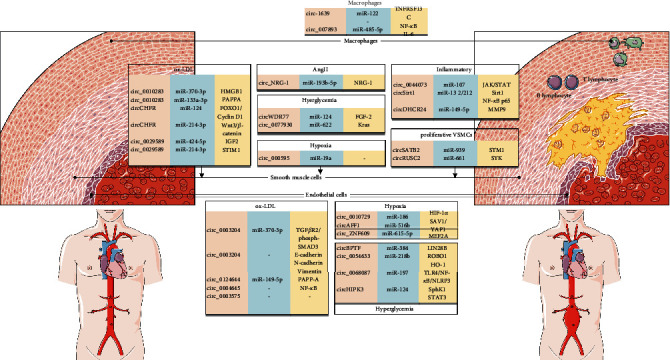
Potential ncRNA in a variety of cells in AAA tissue. Examples of several ncRNAs that may lead to the formation of AAA in macrophages, smooth muscle cells, and endothelial cells include circRNAs, miRNAs, and mRNAs.

At present, the pathogenic mechanism of AAAs is mainly explored through studying aortic tissue from AAA patients undergoing open surgery. Several subtypes of vascular cells (including SMCs, ECs, and adventitial cells) are involved in AAA genesis [20] (Figure 6).

Disordered ECs may induce adventitia and media inflammation [96]. The chronic inflammatory reaction induced by T cells [97] triggers MMP- and macrophage-mediated proteolytic remodeling, finally weakening the ECM structure. Such process is related to elastic fiber loss, collagen fiber structural alterations, and new vessel formation (angiogenesis). Generally speaking, inflammation is a vital contributor to aneurysm occurrence and development. Infiltrating leukocytes and macrophages are the main proteinase sources. The transformation of VSMCs from the contractile/differentiated phenotype to the synthetic/dedifferentiated phenotype correlates with AAA occurrence [98]. VSMCs constitute the principal part of the aortic wall. They proliferate and dedifferentiate to trigger different signaling cascades, which in turn stimulate proliferation, dedifferentiation, apoptosis, and migration of VSMCs.

However, these physiological characteristics facilitate the formation of vulnerable atherosclerotic plaques in the aneurysmal wall, which illustrates that AS is a vital risk factor for AAAs [99]. Age, smoking, and the genetic background are common and the most potent factors leading to these two disorders [100]. Therefore, these processes are at least partly regulated by circRNAs. We aim to speculate the potential relationship of circRNAs with ECs and SMCs in AAAs.

### 5.1. circRNAs and ECs in AAAs

Numerous pathophysiological stimuli can result in EC disorders in AAAs, including hypoxia [101], advanced glycation end products (AGEs) [102], reactive oxygen species (ROS) [103], oxidized low-density lipoproteins (ox-LDLs) [104], and proinflammatory cytokines [105] (Table 2).

A study has revealed the potential role of hypoxia-mediated new vessel formation in the pathogenic mechanism of AAAs [106]. Dang et al. examined the expression patterns of circRNAs in hypoxia-stressed human umbilical vein endothelial cells (HUVECs) and detected the notable upregulation of hsa_circ_0010729. They also found that hsa_circ_0010729 deletion inhibited cell migration and proliferation, but promoted cell apoptosis through targeting the miR-186/HIF-1*α* axis [107]. Recent evidence suggests that the hypoxia-induced circAFF1 can lead to disordered vascular ECs through triggering SAV1/YAP1. From the mechanical perspective, the circAFF1/miR-516b/SAV1/YAP1 axis partially contributes to vascular endothelium dysfunction, and it may be used as an indicator of hypoxia injury-induced vascular diseases [108]. Furthermore, significantly upregulated cZNF609 was observed by Liu et al. in HUVECs exposed to hypoxia and HG environments. As claimed by some researchers, the silencing of cZNF609 prevents EC apoptosis but stimulates tube formation and migration of ECs, demonstrating that cZNF609 can promote the apoptosis of stress-challenged ECs. Mechanically, cZNF609 acts as a ceRNA, which upregulates the expression of myocyte enhancer factor 2A (MEF2A) through separating and suppressing miR-615-5p [109]. According to the above research results, circRNAs regulate EC phenotypes in the hypoxic environment.

High glucose (HG) affects the formation and development of AAAs [110, 111] through enhancing ROS generation [112] and AGE synthesis. In HG-stimulated EC models, circRNAs regulate the phenotypes of ECs. circBPTF is under tight regulation within the HG-challenged HUVECs, and circBPTF deletion prevents the HG-mediated OS and inflammatory injuries through regulating the miR-384/LIN28B axis [113]. In addition, the expression of hsa_circ_0068087 also increases within the HG-challenged HUVECs, and hsa_circ_0068087 deletion attenuates the inflammation and angiogenic disorder in HG-challenged HUVECs through pathways like pyrin domain-containing protein 3 (NLRP3) inflammasome, LRR, and toll-like receptor 4 (TLR4)/NF-*κ*B/NOD as the miR-197 sponge [114]. Previous evidence demonstrates that the expression of hsa_circ_0054633 increases in HG-challenged HUVECs. Downregulating the hsa_circ_0054633 expression blocks HUVEC proliferation, angiogenesis, and migration, but boosts HUVEC apoptosis via the heme oxygenase-1 (HO-1) and miRNA-218/roundabout-1 (ROBO1) axes [115]. In addition, the HG-induced circHIPK3 downregulation enables miR-124 to accumulate in human aortic endothelial cells (HAECs) and HUVECs. From the mechanical perspective, circHIPK3 silencing enhances the HG-mediated apoptosis via the miR-124/sphingosine kinase 1 (SphK1)/signal transducer and activator of transcription 3 (STAT3) axis [116].

OxLDLs affect the O2•- concentration in several ways. Increasing the O2•- concentration causes the NOS to dephosphorize to produce O2•- [117, 118], inhibits dismutase activities, and curbs O2•- reduction to H_2_O_2_. OxLDLs promote inflammatory cell infiltration into the aortic wall through enhancing the activities of MMP2 [119], chemokines, and adhesion particles [120]. Research has found circ_0003204 is mostly located in the cytoplasm of HAECs, and its expression increases in ox-LDL-challenged HAECs. Overexpressed circ_0003204 restrains the proliferation, tube formation, and migration of ox-LDL-challenged HAECs. Mechanically, circ_0003204 acts as the miR-370 sponge, which increases the protein levels of transforming growth factor (TGF) *β*R2 and the corresponding downstream phosph-SMAD3 [121]. A recent study focusing on circ_0003204 underlines the vital role of hsa_circ_0003204 in HUVEC proliferation and new vessel formation. Besides, hsa_circ_0003204 deletion evidently downregulates the expression of E-cadherin and upregulates the expression of vimentin and N-cadherin in oxLDL-challenged HUVECs [122]. Wang et al. have noticed that circ_0124644 alleviates the endothelial injuries in ox-LDL-challenged HUVECs through the miR-149-5p/PAPP-A axis [123]. It is also shown that circ_0003645 deletion mitigates the apoptosis and inflammation in oxLDL-challenged ECs [124].

### 5.2. circRNAs and SMCs in AAAs

VSMC phenotypic transformation within AAAs is ascribed to pathophysiological incentives as diverse as ox-LDLs, Ang II, proinflammatory factors, and hyperglycemia (Table 3).

The circ_0010283 level in ox LDL-challenged VSMCs is remarkably higher than that in control VSMCs. The circ_0010283 target is miR-370-3p, which targets HMGB1. As a result, circ_0010283 modulates the level of HMGB1 through miR-370-3p [125]. The high serum expression of circ_0010283 in AS cases and human VSMCs treated with ox-LDLs is confirmed by Feng et al., who report that circ_0010283 modulates the PAPPA level through regulating miR-133a-3p [126]. Similarly, the abnormal expression of circCHFR is also detected in ox-LDL-treated VSMCs. In addition, the circCHFR/miR-370/FOXO1/Cyclin D1 axis is found to play a key role in SMCs, which increases our knowledge of circRNAs in SMCs [127]. As revealed by a study, circCHFR is upregulated in serum of atherosclerosis patients and ox-LDL-stimulated VSMCs. circCHFR controls cell growth, migration, and inflammation via regulating the expression of Wnt3 as a ceRNA of miR-214 in ox-LDL-treated VSMCs [128]. In the study of Yu et al., increased circ_0029589 levels and decreased miR-424-5p levels in ox-LDL-challenged VSMCs demonstrate that circ_0029589 regulates VSMC proliferation and migration possibly via miR-424-5p/IGF2 [129]. Correspondingly, circ_0029589 deletion suppresses the proliferation, invasion, and migration of ox-LDL-challenged VSMCs through modulating STIM1 and miR-214-3p [130].

Ang II has been suggested to enhance VSMC proliferation in the process of vascular remodeling and play a vital part in AAA formation. According to circRNA microarray analysis, mmu-circRNA-42742 is conspicuously downregulated in Ang II-treated mouse aortic smooth muscle cells (MASMCs). The parental gene of mmu-circRNA-42742 is NRG-1, which modulates vascular remodeling via the ErbB signal transduction pathway. Besides, the circNRG-1/miR-193b-5p/NRG-1 axis can be potentially used as the Ang II target for inhibiting VSMC apoptosis and facilitating vascular remodeling [131].

The analysis of circRNA expression in the HG-induced VSMCs shows that circWDR77 is upregulated. The predicted results are miR-124 and fibroblast growth factor 2 (FGF-2), whose interaction is verified by the RNA pull-down experiment and luciferase reporter gene assay. More importantly, Wang et al. found that exosomes from HG-induced HUVECs caused VSMC senescence via the circRNA-0077930/miR622/Kras ceRNA axis, while exosomes with circRNA-0077930 depletion had no implications for VSMC senescence [132].

Furthermore, Zheng et al. noticed the upregulation of hsa_circ_000595 in the hypoxia-induced human aortic smooth muscle cells (HASMCs). They concluded that hsa_circ_000595 silencing acted as the miR-19a sponge to suppress cell apoptosis [95].

AAAs are a type of chronic inflammatory disorders, so the in vitro AS model can be constructed by inflammatory stimulation. circRNA-0044073 is upregulated in the LPS-treated human umbilical vein smooth muscle cells (HUVSMCs) but downregulated in LPS-treated HUVECs. Further exploration indicates that circRNA-0044073 overexpression evidently promotes HUVSMC and HUVEC proliferation and invasion through the miR-107/Janus kinase (JAK)/STAT signal transduction pathway [133]. As demonstrated by Kong et al., the circ-Sirt1 expression level is distinctly downregulated in TNF-*α*-treated VSMCs, and circ-Sirt1 overexpression impedes the NF-*κ*B p65 nuclear translocation and lowers the levels of adhesion molecules and proinflammatory cytokines (particularly VCAM-1, MCP-1, and ICAM-1) [134]. Moreover, they have also discovered that circ-Sirt1 prevents the transcription and acetylation of NF-*κ*B p65 induced by TNF-*α* through reversing the inhibition effect of miR-132/212 on SIRT1. The study results of Kong et al. suggest that circ-Sirt1 may mitigate the NF-*κ*B p65-induced inflammation via direct and indirect approaches. After treating the human aortic vascular smooth muscle cells (HA-VSMCs) with PDGF-BB, Peng et al. found that circDHCR24 deletion served as the miR-149-5p sponge and diminished the cell proliferation [135].

Additionally, the levels of circ_RUSC2 and circ_SATB2 decrease within the proliferating VSMCs. It is disclosed by Mao et al. that circ-SATB2 promotes STIM1, which can be explained by the role of miR-939 in proliferative VSMCs. Mechanically, circ-SATB2 overexpression inhibits the expression of contractile VSMC marker SM22a, while miR-939 promotes its expression, indicating an association between circ-SATB2 and VSMC phenotypic differentiation [136]. circ_RUSC2 is reported to enhance the proliferation, migration, and phenotypic regulation of VSMCs, but suppress their apoptosis through the miR-661/spleen-associated tyrosine kinase (SYK) signal transduction pathway [137].

### 5.3. circRNAs and Macrophages in AAAs

CircRNAs are proven to be essential for the modulation of macrophages. Studies have shown that in LPS-induced macrophages Raw264.7, circ_1639 promotes the proinflammatory response in Raw264.7 cells. The study of Lu et al. demonstrates that circ_1639 expression is tremendously upregulated in LPS-treated Raw264.7 cells. The level of p-P65 increases in circ_1639 mock-transfected cells, but declines in cells transfected with circ_1639 inhibitor plasmid. Therefore, circ_1639 regulates inflammatory response through the NF-*κ*B signaling pathway. When Raw264.7 cells are transfected with the circ_1639 mimic, the level of miR-122 gene is significantly reduced, while the expression of its target gene TNFRSF13C increases. Hence, it can be concluded that in Raw264.7 cells, circ_1639 affects the inflammatory response through the miR-122/TNFRSF13C regulatory axis [138].

In Raw264.7 macrophages stimulated by the calcitonin gene-related peptide (CGRP), increased expression of mmu_circRNA_007893 and decreased expression of miR-485-5p are detected. The coimmunoprecipitation reaction further confirms the interaction among mmu_circRNA_007893, miR-485-5p, and IL-6. In Raw264.7 cells, the mmu_circRNA_007893/miR-485-5p/IL-6 regulatory pathway regulates the inflammatory response by controlling the expression of the cytokine IL-6 and ultimately affects the cell function [139].

The above results indicate that the function of macrophages can be affected by the expression level of the circRNA regulatory axis, such as the proinflammatory cytokines (e.g., TNF-*α* and IL-6) and anti-inflammatory cytokines. There is a lot of evidence that circRNAs play a vital role in macrophages, and it will assist with further research on the diseases caused by macrophages and circRNAs.

### 5.4. circRNAs in Aortic Dissection Tissue and Intracranial Aneurysms

#### 5.4.1. circRNAs in Aortic Dissection Tissue

In a previous study, a targeted circRNA array was applied to exploring differentially expressed circRNAs in tissue specimens from thoracic aortic dissection (TAA) patients undergoing surgery [140]. As observed from the qRT-PCR assays, the expression levels of hsa_circRNA_102771, hsa_circRNA_002271, hsa_circRNA_101238, hsa_circRNA_104349, hsa_circRNA_104634, COL6A3, and COL1A1 increase, while the expression of hsa_circRNA_005525, hsa_circRNA_102683, hsa_circRNA_103458, and FLNA is downregulated. Meanwhile, circRNA_101238 is found to not only be deregulated with the disease but also potentially affect miR-320a expression and MMP9 levels. Moreover, the expression of both hsa_circRNA_104634 interacting with hsa-miR-145-3p and hsa_circRNA_104349 interacting with hsa-miR-26a-3p is upregulated, which promotes the apoptosis or phenotypic transformation of SMCs [141]. According to another study, hsa_circRNA_104033 and hsa_circRNA_102683 can suppress hsa-miR-195-3p and hsa-miR-29b-1-5p levels, respectively, thereby aggravating aortic wall apoptosis and ECM degradation and promoting collagen remodeling [142]. Thus, those differentially expressed circRNAs discovered possibly contribute to TAD occurrence through several biological processes [140].

Through carrying out RNA-Seq on the affected ascending aortic samples from patients with acute Stanford type A aortic dissection (AAAD), Tian et al. have identified 506 evidently differentially expressed circRNAs [143]. Besides, the levels of ten circRNAs with the most significant differential expression are either increased or decreased by 2-5 folds. Specifically, circUBA2, circARHGAP26, circIQGAP1, circCHSY1, circMED13, circMBNL1, circMYH10, and circRAB7A are upregulated, while circFAM120B and circCEP70 are downregulated. Moreover, the analysis results of the circRNA-miRNA-mRNA network disclose the regulatory effect of circMARK3 on the expression of Fgr, which is a kind of tyrosine-protein kinase. The findings of Tian et al. demonstrate the clinical significance of the circMARK3-miR-1273-Fgr interaction and that the combined use of circRNAs and additional biomarkers can improve the diagnostic accuracy.

#### 5.4.2. circRNAs in Peripheral Blood of Intracranial Aneurysms

One recent effort identifies the hsa_circ_0021001 in peripheral blood of patients with intracranial aneurysms, but its potential contribution to aneurysmal expansion is not expounded [144]. Hsa_circ_0021001 has an area under the receiver operating characteristic ROC curve (AUC) of 0.87, demonstrating its effectiveness in IA diagnosis.

In a study, the circRNA sequencing on IA patients recognizes two novel circRNAs in the peripheral blood samples from IA cases, and their expression in peripheral blood of normal subjects are also analyzed. The results suggest that hsa_circ_0008433 and hsa_circ_0072309 are new and critical circRNAs associated with IAs. This study may provide novel prognostic biomarkers and therapeutic targets for IAs [145].

#### 5.4.3. Hsa_circRNA_0020397 in Intracranial Aneurysm Tissue

Wang et al. acquired arterial wall tissue samples from the aneurysm site in 12 cases and discovered that circRNA_0020397 was downregulated in IAs. The decreased circRNA_0020397 expression possibly suppressed the proliferation of VSMCs through upregulating miR-138 levels and downregulating KDR levels [146].

## 6. Summary and Perspectives

AAAs are one of the major causes leading to cardiovascular death among the senile male population, and their etiology is complex, including apoptosis of SMCs and inflammatory reaction. It is of urgent need to develop new pharmacological approaches or gene therapy strategies for delaying aneurysm development or lowering the risk of acute rupture. Over the last few decades, ncRNAs are increasingly identified as critical regulators for AAA occurrence and development. Hence, it is necessary to identify abnormally expressed ncRNAs and validate them in relevant human AAA tissue and animal models, so as to better explore the pathophysiological mechanisms related to AAA genesis and development.

Different from traditional linear RNAs, circRNAs are a novel class of RNAs with a closed loop structure that can be detected in the eukaryotic genome. Besides, they show higher stability and greater resistance to RNase degradation, so they are widely used as biomarkers. However, there is no evidence that peripheral blood circRNAs are effective biomarkers for the diagnosis of AAAs, and further investigation is required to confirm the relationship between circulating circRNAs and AAAs.

It is reported that circRNAs function as the molecular “sponge,” capable of regulating transcription and posttranscriptional gene expression by binding to and blocking microRNA regulatory factors. The regulatory pathway of circRNAs is circRNA-miRNA-mRNA. A circRNA specifically binds to an miRNA and inhibits its expression, thereby regulating the expression level of the template RNA.

In this paper, the relationship between circRNAs and AAAs is briefly described. It is proved that several circRNAs affect the formation of AAAs by regulating the proliferation and apoptosis of VSMCs. Meanwhile, the changes of aneurysmal wall cells (e.g., immune cells and ECs) in the AAA development process can be well analyzed based on the circRNAs extracted from these cells. When the differential expression of these circRNAs is verified, the molecular mechanisms of circRNAs in regulating AAA occurrence are investigated both in vitro and in vivo. Meanwhile, the mechanism of action is further elaborated.

Because of the above-mentioned characteristics, circRNAs have been listed as a biomarker and therapeutic target for AAAs. The expression and regulation of circRNAs will directly affect the development of AAAs.

## Figures and Tables

**Table 1:** Verified circRNAs in AAAs.

	circRNA	Gene symbol	Regulation	Pathway	Cell type	Function	Ref
*Homo sapiens*	hsa_circ_0002168	TMEM189	Down	hsa-miR-298	circRNA microarray analysis of AAA tissue and healthy aorta tissue	N/A	83
				hsa-miR-503b-5p			
				hsa-miR-296b-3p			
				hsa-miR-612			
				hsa-miR-15a-3p			
				hsa-miR-15a			
	hsa_circ_0005360	LDLR	Down	hsa-miR-670-3p	circRNA microarray analysis of AAA tissue and healthy aorta tissue	N/A	83
				hsa-miR-181b			
				hsa-miR-671-5p			
				hsa-miR-616-3p			
	hsa_circ_0060063	UQCC1	Up	hsa-miR-135b-3p	circRNA microarray analysis of AAA tissue and healthy aorta tissue	N/A	83
				hsa-miR-186-5p			
				hsa-miR-223-3p			
				hsa-miR-541-5p			
				hsa-miR-145b-3p			
	hsa_circ_0070382	AFF1	Up	hsa-miR-137	circRNA microarray analysis of AAA tissue and healthy aorta tissue	N/A	83
				hsa-miR-432-3p			
				hsa-miR-890			
				hsa-miR-135b-3p			
				hsa-miR-24-3p			
	hsa_circ_0027446	HMGA2	Up	hsa-miR-6882-3p	circRNA microarray analysis of AAA tissue and healthy aorta tissue	N/A	83
				hsa-miR-129-5p			
				hsa-miR-331-3p			
				hsa-miR-1236-3p			
				hsa-miR-3925-3p			
	hsa_circ_0028198	ANAPC7	Up	hsa-miR-493-3p	circRNA microarray analysis of AAA tissue and healthy aorta tissue	Expression of ASPH and PDE3B may be regulated by hsa_circ_0028198,	83
				hsa-miR-153-5p			
				hsa-miR-7-5p			
				hsa-miR-205-3p			
	hsa_circ_CCDC66	CCDC66	Up	^∗^hsa-miR-342-3p	SMC	VSMC growth and apoptosis	91
	hsa_circ_CBFB	CBFB	Up	^∗^hsa-miR-28-5p/GRIA4/LYPD3 axis	VSMC	VSMC apoptosis and growth	92
	hsa_circ_CDR1as	CDR1as	Down	^∗^hsa-miRNA-7/CKAP4 axis	AAA and VSMC	VSMC apoptosis and growth	94
	has_circ_000595	N/A	Up	^∗^hsa-miRNA-19a			95

*Mus musculus*	mmu_circ_29892	Pros1	Up	mmu-miR-212-5p	circRNA microarray analysis of Ang II-induced in mice	N/A	90
				mmu-miR-7033-3p			
				mmu-miR-23b-3p			
				mmu-miR-6947-3p			
				mmu-miR-541-5p			
	mmu_circ_45403	Wdr44	Up	mmu-miR-7235-3p	circRNA microarray analysis of Ang II-induced in mice	N/A	90
				mmu-miR-141-5p			
				mmu-miR-7026-3p			
				mmu-miR-301a-5p			
				mmu-miR-92a-2-5p			
	mmu_circ_40388	Kdm3a	Up	mmu-miR-6922-3p	circRNA microarray analysis of Ang II-induced in mice	N/A	90
				mmu-miR-3078-3p			
				mmu-let-7c-1-3p			
				mmu-miR-500-5p			
				mmu-miR-19b-2-5p			
	mmu_circ_27294	Ccdc66	Up	mmu-miR-7092-3p	circRNA microarray analysis of Ang II-induced in mice	N/A	90
				mmu-miR-6946-3p			
				mmu-miR-7094b-2-5p			
				mmu-miR-7578			
				mmu-miR-26a-2-3p			
	mmu_circ_001265	Chordc1	Down	mmu-miR-107-5p	circRNA microarray analysis of Ang II-induced in mice	N/A	90
				mmu-miR-103-1-5p			
				mmu-miR-103-2-5p			
				mmu-let-7a-2-3p			
				mmu-miR-199b-3p			
	mmu_circ_018331	Hivep1	Down	mmu-miR-149-5p	circRNA microarray analysis of Ang II-induced in mice	N/A	90
				mmu-miR-204-5p			
				mmu-miR-30b-3p			
				mmu-miR-7679-5p			
				mmu-miR-5620-5p			
	mmu_circ_40599	Frmd4b	Down	mmu-miR-337-3p	circRNA microarray analysis of Ang II-induced in mice	N/A	90
				mmu-miR-320-5p			
				mmu-miR-7079-5p			
				mmu-miR-215-3p			
				mmu-miR-7021-3p			

**Table 2:** CircRNAs causing EC disorder in AAAs.

circRNA	Cell type	Inducing factor	Expression	Pathway	Function	Ref
circ_0003204	HAEC	ox-LDL	Up	miR-370-3p/TGF*β*R2/phosph-SMAD3	Inhibited viability, migration, proliferation, and tube formation	121
circ_0124644	HUVECs	ox-LDL	Up	miR-149-5p/PAPP-A	Inhibited apoptosis	123
circ_0003645	HUVECs	ox-LDL	Up	NF-*κ*B	Promoted inflammatory response and promoted the production of adhesion molecules	124
hsa_circ_0003575	HUVECs	ox-LDL	Down	—	Promoted apoptosis, inhibited proliferation and angiogenesis	116
circ_0003204	HUVECs	ox-LDL	Down	—	Inhibited the proliferation, migration, and invasion, promoted apoptosis	122
circHIPK3	HUVECs+HAECs	HG	Down	miR-124S/phK1 and STAT3	Inhibited apoptosis	116
circ-0054633	HUVECs	HG	Up	miR-218/roundabout1	Promoted proliferation and migration, inhibited apoptosis	115
				miR-218/heme oxygenase-1		
hsa_circ_0068087	HUVECs	HG	Up	mi-197/TLR4/NF-*κ*B/NLRP3	Promoted inflammation and dysfunction	114
circBPTF	HUVEC	HG	Up	miR-384/LIN28B	Promoted apoptosis, the release of proinflammatory cytokines and oxidative stress	113
hsa_circ_0010729	HUVECs	Hypoxic	Up	miR-186/HIF-1*α*	Promoted proliferation and migration; inhibited apoptosis	107
circAFF1	HUV-EC-C and HBEC-5i	Hypoxic	Up	miR-516b/SAV1/YAP1	Inhibited the proliferation, tube formation, and migration of vascular endothelial cells	108
circ-ZNF609	HUVECs	Hypoxic+HG	Up	miR-615-5p/MEF2A	Promoted apoptosis, inhibited migration and tube formation	109

**Table 3:** CircRNAs causing VSMC disorder in AAAs.

circRNA	Cell type	Inducing factor	Expression	Pathway	Function	Ref
circ_0010283	VSMCs	ox-LDL	Up	miR-370-3p/HMGB1	Promoted VSMC viability and migration, and proliferating cell nuclear antigen	125
	HVSMCs	ox-LDL	Up	miR-133a-3p/PAPPA	Promoted VSMC proliferation, migration and invasion	126
circCHFR	VSMCs	ox-LDL	Up	miR-370/FOXO1/Cyclin D1	Promoted proliferation and migration ability of VSMCs	127
	VSMCs	ox-LDL	Up	miR-214-3p/Wnt3/*β*-catenin	Promoted cell growth, migration, and inflammation	128
circ_0029589	VSMCs	ox-LDL	Up	miR-424-5p/IGF2	Promoted proliferation, migration, and invasion	129
	VSMCs	ox-LDL	Up	miR-214-3p and STIM1	Promoted proliferation, migration, and invasion	130
circNRG-1	MASMCs	Ang II	Down	miR-193b-5p/NRG-1	Promoted apoptosis	131
circWDR77	VSMCs	HG	Up	miR-124/FGF-2	Promoted migration and proliferation	
circ_0077930	HUVEC-Exos>VSMCs	HG	—	miR-622-Kras	Induce cellular senescence in VSMCs	132
circ_0044073	HUVEC	LPS	Down	miR-107/JAK/STAT	Promoted the proliferation and invasion	133
	HUVSMCs		Up			
circ-Sirt1	VSMCs	TNF-*α*	Down	miR-132/212/SIRT1	Inhibited inflammation	134
circDHCR24	HA-VSMC	PDGF-BB	Up	miR-149-5p/MMP9	Promoted proliferation, migration, and phenotypic switch	135
circ-SATB2	Proliferative VSMCs	N/A	Up	miR-939/STM1	Promoted migration, proliferation; inhibited apoptosis	136
circ-RUSC2	Proliferative VSMCs	N/A	Up	miR-661/SYK	Promoted migration, proliferation; inhibited apoptosis	137
circACTA2	HASMCs	N/A	Up	NRG-1/circACTA2/miR-548f-5p	Promoted contraction	

## Data Availability

No data were used to support this study.
